# Editorial: Study of pancreatic islets based on human models to understand pathogenesis of diabetes

**DOI:** 10.3389/fendo.2022.1128653

**Published:** 2023-01-09

**Authors:** Yumi Imai, Scott A. Soleimanpour, Jeffery S. Tessem

**Affiliations:** ^1^ Division of Endocrinology and Metabolism, Department of Internal Medicine, University of Iowa, Iowa City, IA, United States; ^2^ Fraternal Order of Eagles Diabetes Research Center, University of Iowa, Iowa City, IA, United States; ^3^ Endocrinology Section, Iowa City Veterans Affairs Medical Center, Iowa City, IA, United States; ^4^ Department of Internal Medicine, Division of Metabolism, Endocrinology, and Diabetes, University of Michigan, Ann Arbor, MI, United States; ^5^ Department of Nutrition, Dietetics and Food Science, Brigham Young University, Provo, UT, United States

**Keywords:** type 1 diabetes, type 2 diabetes, stem cell derived beta cell-like cells, islet transplant, organoid, micro physiological system, intravital imaging, micro tissue

The central pathology of diabetes mellitus (DM) is failure of pancreatic islets to meet the body’s demand for insulin. Beta cells are lost to autoimmunity in type 1 diabetes (T1D) (1), while increased demand for insulin and nutritional stress cause demise of beta cells in type 2 diabetes (T2D) (2). To develop therapeutics that restore islet function, we need models that recapitulate features of human islets in health and disease states. Animal models, especially genetically modified mice, have increased our understanding of islet physiology. However, human islets differ from mouse islets in many aspects including structure, replication, and stress responses (3). In recent years, new islet models have been developed utilizing human tissues, stem cell derived beta cell-like cells (sBC), and bioengineering technologies. This Research Topic aims to increase the awareness of human cell-based model systems. Here, we discuss three review and two original research articles in this Research Topic that collectively define the status quo of human cell-based islet models and a roadmap for developing newer models in the future.

To model human islets, it is imperative to understand the characteristics of human islets and islet niche. Patel et al. review islet structure and surrounding extracellular matrix as a blueprint to recreate the islet niche. Their review compares currently available models for islet research such as animals, human islets, sBC, and pancreatic slices. Finally, the review summarizes the recent advances in two types of artificial islet niches, 3D-hydrogel and microphysiological systems (MPS, AKA tissue chips, organs-on chips). MPS perfuses cells/islets in a controlled environment to mimic the islet niche *in vivo* and can incorporate assays for functional evaluation of cells/islets. The critical importance of collaborations between biologists and engineers to design MPS tailored to answer particular questions is discussed.

The review by Lorberbaum et al. discusses the importance of human cell-based models using GATA6 as an example. The review provides detailed comparison of animal models and sBC. GATA6 is a transcription factor whose mutations in humans are associated with pancreatic agenesis, neonatal diabetes, and T2D. Interestingly, pancreas-specific GATA6 deletion in mice does not affect pancreas development, possibly due to compensation from the transcription factor GATA4. In contrast, sBC carrying mutant GATA6 are not compensated by GATA4 and show the impairment in differentiation into beta-like cells. However, defects seen in sBCs are milder than expected from affected humans indicating that the current model is not perfect. As a future direction, the review discusses the development of islet organoids consisting of multiple cell types to recreate the entirety of the tissue environment and cell-cell communication.

Transplant of human islets or sBC to animal models place islets/cells in the *in vivo* milieu. The review article by Wagner et al. discusses intravital imaging of human islets transplanted into mice as a technique to assess dynamic changes in human islets *in vivo*. The review describes microscopic techniques, sites appropriate for human islet transplantation with intravital imaging, and mouse models for islet xenotransplants. With the advancement in fluorescent probes, intravital imaging has been successfully used to monitor immune cell infiltration, calcium flux, redox status, and vasculature in islets *in vivo*.

Although human islets from organ donors are often considered the gold standard, they are not a robust model due to heterogeneity in size and composition within the same donor islet preparation, donor to donor variations, and gradual decline in viability in culture. Dispersed human islet cells can be reaggregated into size-controlled organoids termed islet microtissues (MT, AKA pseudoislets) that retain many features of human islets and maintain insulin secretion during long-term culture (4). The research article by Title et al. reports a MT based platform that assesses beta cell function and proliferation with high efficiency. Using tissue clearing technique, they quantified EdU (proliferating) and NKX6.1 (differentiated beta cell) positive nuclei in MTs in a microplate. The platform also measured insulin secretion, insulin content, ATP levels, and caspase 3/7 activity from the same donor islet preparation. The utility of the platform was demonstrated by testing effects of harmine, a highly potent DYRK1A inhibitor known to increase human beta cell replication.

Transplanting iPSC derived sBC to humans circumvents rejection from alloimmunity. However, sBC are still at risk of autoimmune attack when transplanted into T1D subjects. To formulate a strategy to protect sBC transplanted into T1D subjects, we need an animal model to assess autoimmunity against sBC *in vivo*. The humanized mouse model created by adoptive transfer of human immune cells does not develop autoimmune insulitis unless engrafted by human immune cells that are autoreactive to beta cells. Also, adoptively transferred human immune cells do not traffic efficiently to the islet graft site. To overcome limitation, Santini-González et al. transplanted sBC clusters bearing a specific HLA class I (sBCs HLA-A*02:01) under kidney capsule of NOD mice that express matching human HLA class I. CD3^+^ T cells showed massive infiltration at the site of transplant, histology compatible with autoimmune insulitis. Moreover, knockdown of PD-L1 and all HLA class I molecules in sBC improved the survival of sBC graft, supporting the possibility to induce immune tolerance by modulating sBC. In addition to autoimmunity, allo- and xeno-immunity may contribute to insulitis in the model. However, the study is an important step forward to establish a model in which autoimmunity against sBC can be tested *in vivo*.

This collection of articles displays the power of new models based on human cells to dissect the pathophysiology of pancreatic islet dysfunction in DM. We likely will see further refinement of models, especially for sBC, organoids, and MPS in the years to come. In addition, researchers now have access to data collected from human islets with the Human Islet Research Network (HiRN, https://hirnetwork.org/resources), Translational Human Pancreatic Islet Genotype Tissue-Expression Resource (TIGER, http://tiger.bsc.es), Alberta Islet Core (https://www.epicore.ualberta.ca/isletcore) and others. We are in an exciting era in which the pathogenesis of islet failure in DM can be addressed utilizing tools with high translational value, which ultimately could lead to new therapies for DM.

## Author contributions

YI, SS, and JST conceptualized and wrote the manuscript. All authors approved the final version of the manuscript.
